# Regulatory Effects of Senescent Mesenchymal Stem Cells: Endotheliocyte Reaction

**DOI:** 10.3390/cells13161345

**Published:** 2024-08-13

**Authors:** Andrey Ratushnyy, Mariia Ezdakova, Diana Matveeva, Ekaterina Tyrina, Ludmila Buravkova

**Affiliations:** Institute of Biomedical Problems, Russian Academy of Sciences, Khoroshevskoye Shosse, 76a, 123007 Moscow, Russia; ezdakova.mi@gmail.com (M.E.); matveeva.dajana@yandex.ru (D.M.); eagolikovamsu@gmail.com (E.T.); buravkova@imbp.ru (L.B.)

**Keywords:** mesenchymal stem cells (MSCs), ASC52telo, endothelial cells (ECs), mitomycin C (MmC), senescence, SASP

## Abstract

Currently, there is a growing focus on aging and age-related diseases. The processes of aging are based on cell senescence, which results in changes in intercellular communications and pathological alterations in tissues. In the present study, we investigate the influence of senescent mesenchymal stem cells (MSCs) on endothelial cells (ECs). In order to induce senescence in MSCs, we employed a method of stress-induced senescence utilizing mitomycin C (MmC). Subsequent experiments involved the interaction of ECs with MSCs in a coculture or the treatment of ECs with the secretome of senescent MSCs. After 48 h, we assessed the EC state. Our findings revealed that direct interaction led to a decrease in EC proliferation and migratory activity of the coculture. Furthermore, there was an increase in the activity of the lysosomal compartment, as well as an upregulation of the genes *P21*, *IL6*, *IL8*, *ITGA1*, and *ITGB1*. Treatment of ECs with the “senescent” secretome resulted in less pronounced effects, although a decrease in proliferation and an increase in ICAM-1 expression were observed. The maintenance of high levels of typical “senescent” cytokines and growth factors after 48 h suggests that the addition of the “senescent” secretome may have a prolonged effect on the cells. It is noteworthy that in samples treated with the “senescent” secretome, the level of PDGF-AA was higher, which may explain some of the pro-regenerative effects of senescent cells. Therefore, the detected changes may underlie both the negative and positive effects of senescence. The findings provide insight into the effects of cell senescence in vitro, where many of the organism’s regulatory mechanisms are absent.

## 1. Introduction

The phenomenon of aging involves complex, interrelated mechanisms at multiple biological levels. At the organism level, aging leads to a decrease in the ability to maintain homeostasis due to organ dysfunction. At the tissue level, chronic inflammation is observed as a driver of many age-related diseases, including cardiovascular and neurodegenerative pathologies. These pathological transformations are based on changes at the cellular level, expressed in alterations in the qualitative and quantitative cellular composition of tissues and in the disruption of intercellular communication. The ratio between differentiated and progenitor cells changes, and the proportion of senescent cells, whose morphofunctional state is significantly modified, increases [1,2,3,4,5].

In the last decade, more and more attention has been paid to paracrine changes. Besides cell cycle arrest, one of the most characteristic features of senescent cells (and perhaps the most important from the perspective of the aging organism) is the senescence-associated secretory phenotype (SASP). The paracrine profile includes hundreds of secreted factors, including pro-inflammatory cytokines, chemokines, growth factors, and proteases. The specific composition can vary depending on the cell type and the method used to induce senescence. The SASP is a particular but important part of the disruption of intercellular communication, which has many consequences for the surrounding tissues. A growing body of preclinical and clinical research has demonstrated a correlation between senescence, SASP, and age-related vascular pathologies [6,7,8,9].

Cells that play a regulatory role in tissues, such as mesenchymal stem cells (MSCs), may be critical. MSCs are of particular interest for both basic science and applied applications in regenerative medicine. Researchers conclude that the functional state of organs and tissues largely depends on MSCs, which occupy the perivascular niche and are involved in the regulation of angiogenesis, immunomodulation, maintenance of hematopoiesis, etc. [10,11]. MSCs are known to secrete various vasoactive peptides, growth factors, and cytokines that regulate endothelial cell (EC) proliferation, survival, migration, and vascular permeability. The interaction between MSCs and ECs supports the formation and function of the vasculature in vivo. Their angiogenic potential is of particular interest in the treatment of ischemia, where the stimulation of vascularization is critical for the preservation of living tissue and the prevention of fibrosis [12,13,14].

Given their role in maintaining tissue homeostasis and regulating cell-to-cell communication, MSCs are of particular interest in the context of senescence. The fundamental role of adult stem cells in maintaining tissue function is an intriguing area of research. On the other hand, the elderly represent the primary population for cell transplantation therapy; the aging of donors and the subsequent decline in the quantity and quality of MSCs limits the efficacy of autologous MSC transplantation therapy. Therefore, it is necessary to analyze the regulatory potential of senescent MSCs in order to modulate the efficacy of autologous MSC-based therapy, particularly in elderly patients. Like other cells, MSCs change their morphofunctional characteristics upon activation of the senescent state. Irreversible cell cycle arrest is observed, accompanied by morphological, organelle activity, and gene expression changes, as well as the detection of a number of other markers of cellular aging. At the same time, MSCs continue to interact with their environment, exerting local and systemic influence [15,16,17]. As MSCs occupy a perivascular niche, their closest neighbors are the vascular endothelium. Thus, the objective of the present study was to investigate the impact of senescent MSCs on the functional status of endothelial cells.

In our research, we employed immortalized standard cell lines to mitigate the influence of individual donors’ diversity and enhance the replicability of our findings. The in vitro model enables us to examine specific cell-to-cell interactions independently of the regulatory systems of the organism. As with any other in vitro model, our model does not permit us to ascertain what is occurring within the organism; however, it does reveal some patterns of fundamental intercellular communications. Moreover, our findings contribute to the expansion of knowledge about the physiology of standard cell lines that are frequently employed in research.

## 2. Materials and Methods

### 2.1. Cell Culture

Linear hTERT-immortalized adipose-derived mesenchymal stem cells (ASC52telo—ATCC^®^ SCRC-4000™) and ECs (EA.hy926—ATCC^®^ CRL-2922™) obtained from Lomonosov Moscow State University were used in this study. For cultivation, we used DMEM/F12 medium (Gibco, Life Technologies, Carlsbad, CA, USA) with the addition of 10% fetal calf serum (HyClone, Logan, UT, USA), 100 U/mL penicillin (PanEco, Moscow, Russia), and 100 µg/mL streptomycin (PanEco, Moscow, Russia). MSCs and ECs were continuously cultured under standard laboratory conditions in a CO_2_ incubator (Sanyo, Japan). Cells were passaged at 80–90% confluency. The linear cells were characterized. MSCs were stained with fluorescent antibodies against stromal markers CD90, CD73, CD105, CD29, HLA-ABC, and CD54 (BD Biosciences, San Jose, CA, USA). Fluorescence was analyzed using an Accuri C6 flow cytometer (BD Biosciences, San Jose, CA, USA). The phenotype of EA.hy926 was characterized by staining with antibodies antiCD31-PE and anti-CD144-FITC (BD Biosciences, San Jose, CA, USA). Confirmation of the endothelial nature was the ability to create capillary-like tubes on matrigel. To do this, we used a model for the formation of a capillary-like tube in a “Matrigel” (Corning, NY, USA) in accordance with the manufacturer’s protocol. A total number of tubular complexes was counted with Image Processing Software—Image-Pro Plus 7.0 (Media Cybernetics, Inc., Rockville, MD, USA).

### 2.2. Mitomicyn C (MmC) Treatment

Mitomycin C (MmC) was used to obtain stress-induced senescent MSCs. A Petri dish was seeded with 250,000 cells. Cells (90–100% confluence) were incubated in a complete growth medium containing 1.5 μg/mL MmC for 18 h and then washed twice with PBS and cultured for 10 days without reseeding. MSCs without MmC served as the control (MSCs/MmC−).

### 2.3. Cell Senescence Identification

Expression of senescence associated-β-galactosidase (SA-β-gal) activity at pH 6.0 was estimated with the Senescence Cells Histochemical Staining Kit (Sigma, St. Louis, MO, USA) following the manufacturer’s protocol. We analyzed cell cultures using an Eclipse TiU phase-contrast microscope (Nikon, Japan).

For cell size and structure analysis, flow cytometric forward scatter (FSC) and side scatter (SSC) density plots were applied (Accuri C6 flow cytometer, BD Biosciences, San Jose, CA, USA). The enlargement of cells and increase of the granularity are considered specific features of cell senescence as well [18,19].

Autofluorescence was used as a fast and non-invasive senescence assay [20] via the Accuri C6 flow cytometer (BD Biosciences, San Jose, CA, USA). Data were acquired using the excitation laser at 488 nm and detection optic at 533/30 nm.

For fibroblastic colony forming unit (CFU-f) evaluation, MSCs were plated at a limiting density—100 cells per 60 mm cell culture dish (Corning, NY, USA). After 10 days, the dishes were stained with 0.5% crystal violet solution for 5 min (Sigma-Aldrich, Saint Louis, MO, USA). CFU-fs were analyzed using an Eclipse TiU microscope (Nikon, Japan).

### 2.4. MSC-EC Cocultivation

The system of direct interaction between MSCs and ECs was used in the experiment. MSCs and ECs were seeded in a Petri dish in a 1:1 ratio and cocultured for 48 h. Effects were assessed in the following experimental groups:

- ECs cultivated on plastic in standard growth medium;
- EC(MSC/MᴍC−)—ECs after cocultivation with MSCs (MᴍC−);
- EC(MSC/MᴍC+)—ECs after cocultivation with MSCs (MᴍC+).

After the end of exposure, the conditioned medium was collected for further determination of soluble mediators; cells were washed with phosphate buffer and further analyzed. The coculture was trypsinized and separated using magnetic immunoseparation (Miltenyi Biotec, Bergisch Gladbach, Germany). The CD31+ cells (ECs) were trapped in a magnetic field, resulting in a suspension of unbound cells (MSCs, CD31−) coming out through the pores of the column. After magnetic separation, the cells were collected using centrifugation; the purity of isolation was determined by staining an aliquot of cells with anti-CD31 antibodies, followed by flow cytometry, and the remaining suspension was used for further analysis. To determine the activity level of intracellular compartments, oxidative stress, cell autofluorescence, and viability, we used undivided coculture stained with probes and anti-CD90-APC antibodies to Thy-1 antigen of MSCs, which allowed us to analyze the fluorescence intensity of probes in CD90+ (MSCs) and CD90− (ECs) populations. The cell count of ECs after interaction with MSCs was assessed by cell culture counting using a hemocytometer.

### 2.5. Evaluation of Conditioned Medium Regulatory Effects

To detect the effects of MSC secretome, conditioned medium (CM) from MSCs was added to EC culture medium in a 1:1 ratio. The exposure time was 48 h. Effects were assessed in the following experimental groups:

- ECs cultivated on plastic in a standard growth medium;
- EC(CM/MᴍC−)—ECs cultured on plastic in CM from MSCs (MᴍC−);
- EC(CM/MᴍC+)—ECs cultured on plastic in CM from MSCs (MᴍC+).

After the end of exposure, the CM was collected for further determination of soluble mediators; cells were washed with phosphate buffer, trypsinized, and subjected to further analysis. The cellular increase in endothelial cells after exposure to CM from MSCs was assessed by cell culture counting using a hemocytometer.

### 2.6. Non-Targeted Cell Migration Assay (“Wound Healing”)

The migration of non-targeted ECs or coculture ECs-MSCs was assessed in cell monolayers at a density of 10^4^ cells/cm^2^ using the in vitro “scratch” assay. A sterile pipette tip was used to create a wound approximately 0.8–1.0 mm in width. At the Petri dish, five sections of the “wound” were randomly marked points, which were photographed on a Nikon Eclipse Ti-U microscope immediately after the mechanical injury (0 h point) and after 6 h. The images were analyzed using NIS-Elements software AR 3.10 (Nikon, Tokyo, Japan), which measured the width of the scratch at previously marked points along its length. The percentage of gap closure was calculated using the following formula: (1 − N/N0) × 100%, where N represents the final wound area and N0 denotes the initial wound area.

### 2.7. Flow Cytometry

Morphological parameters, viability, ROS, lysosomal and mitochondrial compartment activity, and analysis of the expression of intercellular interaction molecules were determined on a Cytoflex S flow cytofluorimeter (Beckman Coulter, Indianapolis, IN, USA). The annexin V-propidium iodide test (ANNEXIN V-FITC Kit, Beckman Coulter) was used to assess cell viability. Cells undergoing apoptosis were stained with annexin conjugated to the fluorescent dye FITC, and fluorescent pro pidium iodide was bound to the DNA of necrotic cells. The coculture was stained with anti-CD90-APC antibodies against MSC antigen, allowing viability to be analyzed in CD90+ (MSC) and CD90− (EC) populations. Cells not stained with annexin V and propidium iodide were considered alive. The pH-dependent marker LysoTracker Green DND-26 (Life Technologies, Carlsbad, CA, USA) was used to determine the functional activity of lysosomes. Detection of reactive oxygen species was performed using CM-H2DCFDA (MilliporeSigma, Burlington, MA, USA). The volume of the mitochondrial compartment was estimated using the MitoTracker Red probe (Thermo Fisher Scientific, Waltham, MA, USA). Antibodies to ICAM-1 (CD54) (BD Bioscience, Franklin Lakes, NJ, USA) conjugated with fluorescent tags were used to analyze the expression of intercellular interaction molecules involved in the adhesion of immune cells to the endothelium.

### 2.8. Analysis of Secreted Proteins

The conditioned medium (CM) was collected before reseeding, centrifuged at 2500× *g* to remove cell debris, and stored at −80 °C (low-temperature freezer, Sanyo, Osaka, Japan).

To detect 105 different cytokines, CM was analyzed using Proteome Profiler Human XL Cytokine Array Kit (R&D Systems Inc., Minneapolis, MN, USA) according to the manufacturer’s instructions. The Kit is a membrane-based sandwich immunoassay. Capture antibodies spotted in duplicate on nitrocellulose membranes bind to specific target proteins present in the sample. The data were analyzed using Image Lab Software Version 5.0 (Bio-Rad, Hercules, CA, USA).

Forty-eight cytokines were measured using multiplexed fluorescent bead-based immunoassay detection (MILLIPLEX^®^ MAP system, Merck Millipore, Darmstadt, Germany) according to the manufacturer’s instructions with a Human Cytokine/Chemokine/Growth Factor Panel A—Immunology Multiplex Assay. The panel contained antibody-conjugated beads for following cytokines and chemokines: sCD40L, EGF, Eotaxin, FGF-2, Flt-3 ligand, Fractalkine, G-CSF, GM-CSF, GROα, IFNα2, IFNγ, IL-1α, IL-1β, IL-1ra, IL-2, IL-3, IL-4, IL-5, IL-6, IL-7, IL-8, IL-9, IL-10, IL-12(p40), IL-12(p70), IL-13, IL-15, IL-17A, IL-17E/IL-25, IL-17F, IL-18, IL-22, IL-27, IP-10, MCP-1, MCP-3, M-CSF, MDC (CCL22), MIG, MIP-1α, MIP-1β, PDGF-AA, PDGF-AB/BB, RANTES, TGFα, TNFα, TNFβ, and VEGF-A. For each assay, the curve was derived from various concentrations of the cytokine standards assayed in the same manner as conditioned medium samples. All samples were measured undiluted.

### 2.9. Quantitative PCR Analysis

To evaluate gene expression, total RNA was extracted with ExtractRNA Reagent (Evrogen, Moscow, Russia) and purified using the phenol/chloroform technique. The quality and concentration of RNA samples were estimated by using a Nanodrop ND-2000c (Thermo Scientific, Waltham, MA, USA). Ambion DNase I (RNase-free) (Thermo Fisher Scientific, Waltham, MA, USA) was used for genomic DNA degradation. Reverse transcription was performed using the MMLV RT Kit (Evrogene, Moscow, Russia) according to the manufacturer’s protocol. The expression of 84 cytokine genes was analyzed using RT^2^ Profiler™ PCR Array Human Growth Factors (Qiagen, Hilden, Germany). This gene profiler included *ACTB*, *AMH*, *B2M*, *BDNF*, *BMP1*, *BMP10*, *BMP2*, *BMP3*, *BMP4*, *BMP5*, *BMP6*, *BMP7*, *BMP8B*, *CECR1*, *CLC*, *CSF1*, *CSF2*, *CSF3*, *CSPG5*, *CXCL1*, *DKK1*, *ERAP1*, *EREG*, *FGF1*, *FGF11*, *FGF13*, *FGF14*, *FGF17*, *FGF19*, *FGF2*, *FGF22*, *FGF23*, *FGF5*, *FGF6*, *FGF7*, *FGF9*, *FIGF*, *GAPDH*, *GDF10*, *GDF10*, *GDF11*, *GDNF*, *GPI*, *HBEGF*, *HPRT1*, *IGF1*, *IGF2*, *IL10*, *IL11*, *IL12B*, *IL18*, *IL1A*, *IL1B*, *IL2*, *IL3*, *IL4*, *INHA*, *INHBA*, *INHBB*, *JAG1*, *JAG2*, *LEFTY1*, *LEFTY2*, *LIF*, *LTBP4*, *MDK*, *MSTN*, *NDP*, *NGF*, *NODAL*, *NRG1*, *NRG2*, *NRG3*, *NRTN*, *NTF3*, *OSGIN1*, *PDGFC*, *PGF*, *PSPN*, *PTN*, *RPLP0*, *SLCO1A2*, *SPP1*, *TDGF1*, *TGFB1*, *THPO*, *TNNT1*, *TYMP*, *VEGFA*, and *VEGFC*. The cDNA was mixed with qPCRmix-HS SYBR (Evrogene, Moscow, Russia) and added to 96-well plates according to the manufacturer’s protocol. The expression levels of five housekeeping genes (*ACTB*, *B2M*, *GAPDH*, *HPRT*, and *RPLP0*) were used for reference. The expression of *CDKN2A* (*P16*), *CDKN1A* (*P21*), *hTERT*, *IL-6*, *IL-8*, *ICAM-1*, *ITGA1*, and *ITGB1* was analyzed using Qiagen primers (Qiagen, Hilden, Germany). Polymerase chain reaction was performed using the Mx3000P system (Stratagene, San Diego, CA, USA). Normalized gene expression was calculated with the 2^−ΔCt^ and 2^−ΔΔCt^ methods.

### 2.10. Statistical Analysis

A minimum of three independent experiments were performed for each assay. Analysis of group differences was performed using the nonparametric Mann–Whitney test for independent samples using SPSS 14.0 software (SPSS Inc., Chicago, IL, USA). A level of *p* < 0.05 was accepted as statistically significant.

### 2.11. Graphical Abstract

A graphical abstract was created with BioRender.com (Agreement number: ZB274GD5YT).

## 3. Results

**Figure 1:**
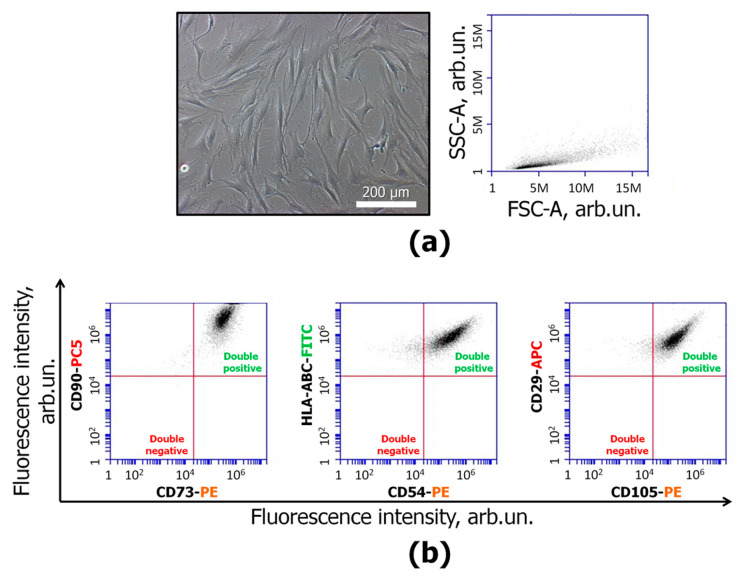
ASC52telo characterization: (**a**) Left—representative image of MSCs with fibroblast-like morphology, phase contrast microscopy, bar-100 µm. Right—SSC-A/FSC-A distribution of MSCs; (**b**) cell surface markers on MSCs. MSCs were positive for CD90-PC5, CD73-PE, HLA-ABC-FITC, CD54-PE, CD105-PE, and CD29-APC. Flow cytometry, representative histograms. *n* ≥ 4.

The work included several main stages: characterization of cell lines, characterization of MmC-treated MSCs, evaluation of MSC-EC cocultivation effects, and evaluation of conditioned medium effects. In the first step, the provided cell lines were characterized. The line of MSCs (ASC52telo) was analyzed. The cells had the ability to adhere to the plastic surface and showed a typical fibroblast-like morphology (Figure 1a). The cells were positive-stained with antibodies against stromal markers CD90, CD73, CD105, CD29, HLA-ABC, and CD54 (Figure 1b). These data indicate that the obtained cells have MSC characteristics [21].

**Figure 2:**
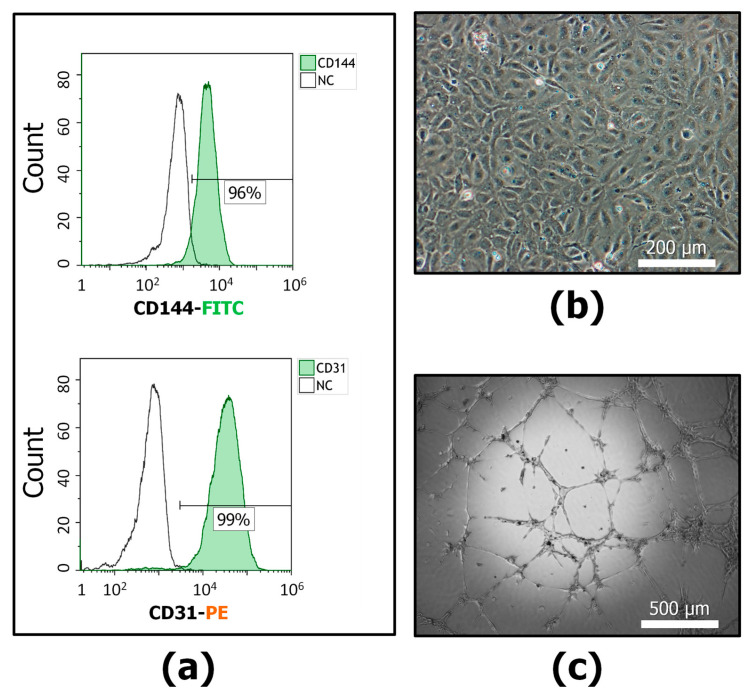
The EA.hy926 (endothelial cell culture, ECs) characterization: (**a**) Representative flow cytometric plots of EC immunophenotype. ECs were positive for CD144-FITC and CD31-PE (green color is positive cells). NC is an unstained control. (**b**) Representative image of ECs, phase contrast microscopy, bar-200 µm. (**c**) Capillary-like structures (tubes) in “matrigel” formed by EA.hy926 cells, phase contrast, bar-500 µm. *n* ≥ 4.

To identify the regulatory features of senescent MSCs, we used a human cell line (EA.hy926) that preserves the characteristics of human umbilical vein endothelial cells. The phenotype was characterized by staining with antibodies against CD31 and CD144 (Figure 2a). The cells were CD144 (VE-cadherin, vascular endothelial cadherin) and CD31 (PECAM-1, platelet endothelial cell adhesion molecule) positive. The ECs had a polygonal and rounded shape characteristic of the endothelium, and as they grew, they covered the entire surface of the dish, fitting tightly to each other and forming a typical monolayer (Figure 2b). Confirmation of the endothelial nature was the ability to create capillary-like tubes on matrigel, which is typical for endothelial cells (Figure 2c). Thus, the cells used exhibited the main characteristics of MSCs and ECs.

### 3.1. Characterization of MmC-Treated MSCs

The senescence of MSCs was induced using mitomycin C (MmC). MmC is an antitumor antibiotic that is commonly utilized in the chemotherapy of malignant neoplasms [22]. Additionally, it is employed in biotechnological research to generate a feeder layer of non-dividing fibroblasts, which serve to support the growth of various stem cells [23]. The effect of MmC on cells is to cause serious DNA damage, which in turn disrupts replication processes and activates stress-induced cell senescence. Higher concentrations lead to significant transcriptional disturbances and apoptosis [24,25]. MmC acts as a bi-alkylating and DNA crosslinking chemotherapeutic agent. Cell senescence is a natural mechanism that serves to prevent the development of oncogenic processes.

The MSCs were treated with MmC for 18 h. Following exposure, the medium was changed, and the cells were cultured for 10 days to allow the cell response to stress and the acquisition of the senescent phenotype to fully manifest. During cultivation, the medium was changed every 3–4 days. After 10 days, the cells were analyzed.

**Figure 3:**
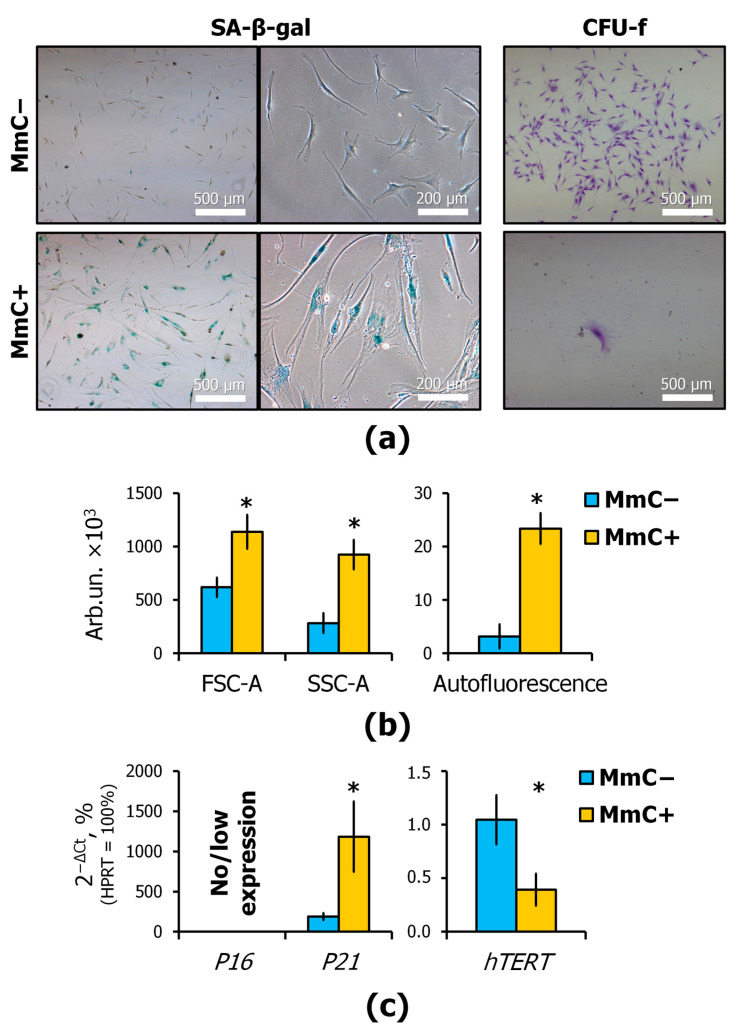
Senescence markers of MmC-treated MSCs: (**a**) histochemical evaluation of senescence associated-β-galactosidase (SA-β-gal) activity and colony-forming unit of fibroblasts (CFU-f) assay, light microscopy; (**b**) average FSC-A (size), SSC-A (cytoplasm vacuolization) and autofluorescence; (**c**) expression of senescence markers *CDKN2A* (*P16*), *CDKN1A* (*P21*), and gene of telomerase reverse transcriptase *hTERT*, q-PCR. Data are shown as mean ± SD; *n* ≥ 4, * *p* < 0.05. MmC—mitomycin C, MSCs—mesenchymal stem cells.

A portion of the cells were subcultured in order to analyze the activity of senescence-associated β-galactosidase (SA-β-gal) and to identify the clonogenic potential of CFU-f (Figure 3a). The SA-β-gal activity was analyzed after 48 h. The number and morphology of colonies were analyzed after 14 days. The remaining portion was assessed using flow cytometry and microscopy. Morphological indicators and autofluorescence were studied (Figure 3b). Additionally, the expression of aging-associated genes, including cyclin-dependent kinase inhibitors (*CDKN2A*, *P16*; *CDKN1A*, *P21*), as well as the gene of telomerase reverse transcriptase (*hTERT*), was analyzed (Figure 3c).

In MmC+ dishes, nearly 100% of the cells exhibited the activity of SA-β-gal, whereas in MmC− dishes, no stained MSCs were observed. This approach to confirming senescence is one of the most widely utilized methodologies in the field. The SA-β-gal is a lysosomal hydrolase that is activated at pH 4. Nevertheless, it has been demonstrated that SA-β-gal may be activated at pH 6 in senescent cells [26].

The principal feature of cell senescence is the stable cessation of cell division. To assess the proliferative activity of the MSCs, the cells were seeded at a low density on new culture dishes (100 cells per 60 mm dish). After 10 days, colony formation was evaluated. It has been demonstrated that treated cells are unable to form new colonies. This observation corroborates the evidence of cell cycle arrest.

MmC-treated MSCs exhibited a flattened and enlarged morphology that deviated from the typical “young” spindle-shaped cells. Some studies have indicated that cell volume enlargement is a consequence of an arrest in cell division. Despite the inhibition of the cell cycle by P21 or P16, macromolecule synthesis can be continually driven by activated PI3K and the mammalian target of rapamycin (mTOR), which causes a sustained enlargement in cell volume without proliferation [27,28]. Another consequence of the inability of cells to divide is the accumulation of lipofuscin, which exhibits strong autofluorescence. A proliferated cell is able to divide undegradable waste material between daughter cells, whereas a senescent cell lacks this opportunity and continues to accumulate lipofuscin. Furthermore, a shift in the balance between the accumulation of damages and autophagic processes contributes to this phenomenon. Consequently, in senescent cells, under the influence of stress, the level of autofluorescence increases due to the aggregation within the cytoplasm of lipofuscin and lipofuscin-like compounds. The analysis of cell autofluorescence can be rapidly and extensively employed, as it is based on common flow cytometry or fluorescent microscopies, which detect the excitation of endogenous fluorophores produced by cell constituents [20,29]. In the course of our experiments, we observed that MSCs treated with MmC exhibited a pronounced autofluorescence.

The activity of the cyclin-dependent kinase inhibitors P21 and P16 plays a pivotal role in the regulation of senescence. The DNA damage response (DDR) is a signaling pathway in which ATM or ATR kinases block the cell cycle progression through the transcriptional activation of P21. However, DDR-independent senescence involves P16 [2,30]. The use of qPCR revealed an increase in the expression of the gene encoding the cyclin-dependent kinase inhibitor P21 (*CDKN1A*). In this case, there was no significant expression of the *P16* gene (*CDKN2A*). It is noteworthy that MmC treatment of immortalized MSCs resulted in a decrease in telomerase (*hTERT*) expression. The phenomenon of senescence is typically attributed to the shortening of telomeres and the lack of telomerase activity. Our findings suggest that sublethal stress may result in a reduction in telomerase expression.

The data collectively demonstrate that MSCs undergo a senescent state following MmC treatment. This process is probably implemented through the P53/P21 signaling pathway.

The SASP is a defining feature of senescent cells. It comprises the release of a multitude of cytokines, chemokines, growth factors, and proteases into the extracellular environment. Senescence can modulate pathways in neighboring cells and tissues through SASP. It is noteworthy that senescent cells induced by disparate stress stimuli may exhibit distinctive SASP components [31].

**Figure 4:**
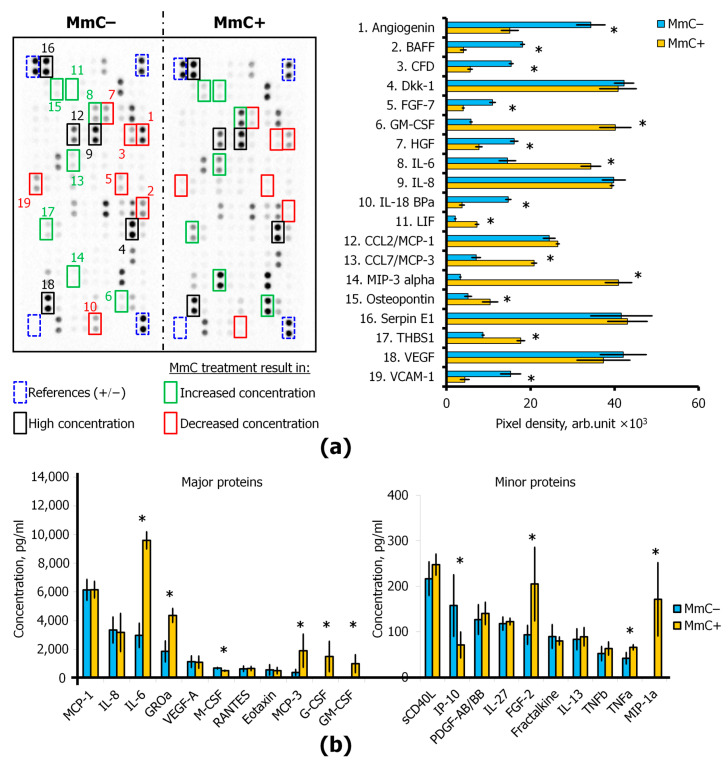
Evaluation of senescence-associated secretory phenotype (SASP) in conditioned medium (CM) of “young” (MmC−) and senescent (MmC+) MSCs: (**a**) representative image of the dot blot (Human XL Cytokine Antibody Array Kit) and protein production. Densitometric analysis of certain analytes in antibody arrays was quantified using ImageLab software 5.0 (Bio-Rad). (**b**) Multiplexed fluorescent bead-based immunoassay detection of cytokines in CM using Human Cytokine/Chemokine (48-plex) panel. Data are shown as mean ± SD; *n* ≥ 4, * *p* < 0.05. MmC—mitomycin C, MSCs—mesenchymal stem cells.

The content of more than 100 different cytokines in the conditioned medium was assessed using dot blots (Human XL Cytokine Antibody Array, R&D Systems Inc., Minneapolis, MN, USA). Treatment with MmC resulted in a more than twofold increase in the level of seven proteins (MIP-3, GM-CSF, LIF, CCL7, IL-6, Thrombospondin-1, and Osteopontin). Concurrently, the concentration of MIP-3 exhibited a more than 10-fold increase. Conversely, the levels of seven proteins (HGF, Angiogenin, Complement Factor D, KGF/FGF-7, VCAM-1, IL-18 BPa, BAFF) were decreased (Figure 4a).

A multiplex analysis was employed for a more detailed quantitative assessment, enabling the detection of the content of 48 cytokines and growth factors. MmC treatment was found to trigger the pronounced production of GM-CSF, G-CSF, and MIP-1a. The concentrations of MCP-3, IL-6, GROa, and FGF-2 were observed to increase by more than twofold. In contrast, M-CSF and IP-10 exhibited a decrease (Figure 4b).

**Figure 5:**
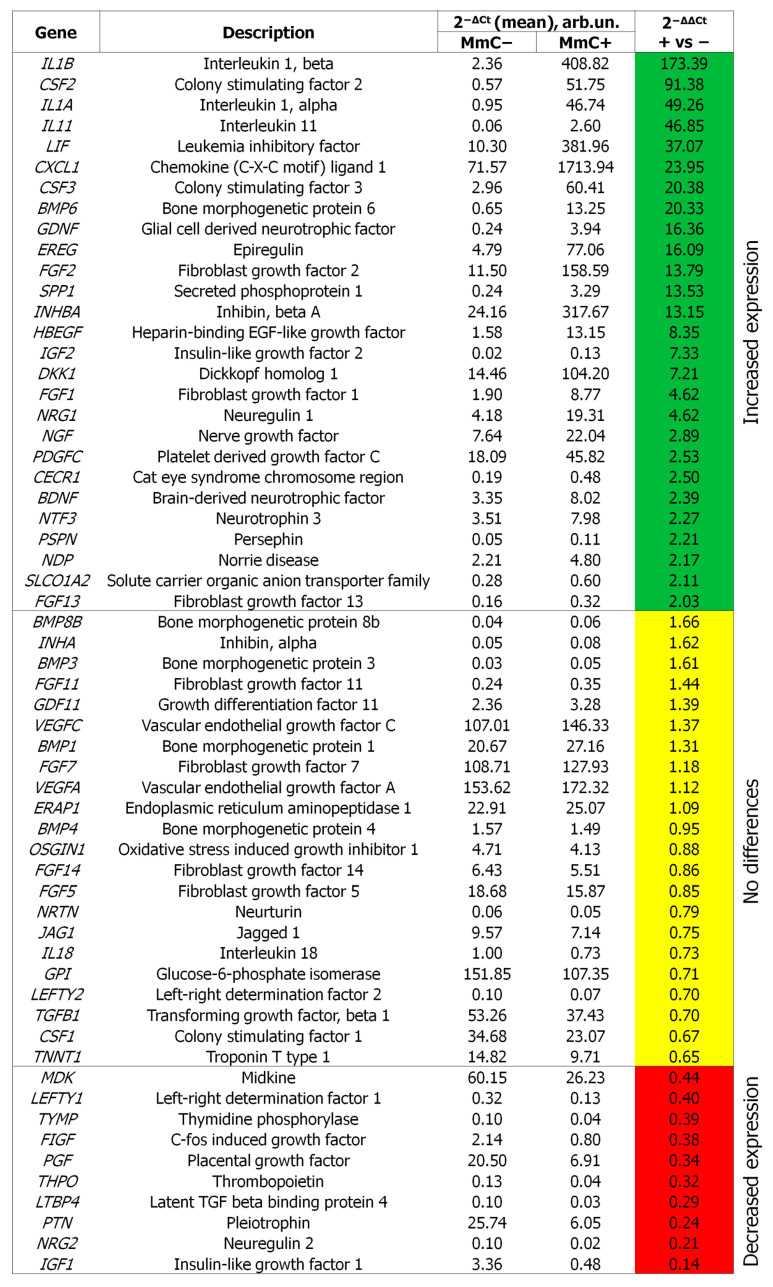
The differential expression of secretome-associated genes in MmC-treated MSCs. Normalized gene expression was calculated with the 2^−ΔCt^ and 2^−ΔΔCt^ methods. Green—significantly upregulated genes, yellow—no differences, red—significantly downregulated genes (*p* < 0.05); *n* ≥ 4. MmC—mitomycin C, MSCs—mesenchymal stem cells.

To analyze changes at the transcriptional level, quantitative PCR analysis was employed. Treatment with MmC resulted in a more than twofold increase in transcriptional activity for 27 genes (*IL1B*, *CSF2*, *IL1A*, *IL11*, *LIF*, *CXCL1*, *CSF3*, *BMP6*, *GDNF*, *EREG*, *FGF2*, *SPP1*, *INHBA*, *HBEGF*, *IGF2*, *DKK1*, *FGF1*, *NRG1*, *NGF*, *PDGFC*, *CECR1*, *BDNF*, *NTF3*, *PSPN*, *NDP*, *SLCO1A2*, *FGF13*) and a reduction in transcriptional activity for 10 genes (*MDK*, *LEFTY1*, *TYMP*, *FIGF*, *PGF*, *THPO*, *LTBP4*, *PTN*, *NRG2*, *IGF1*) (Figure 5). The results of the transcriptomic analysis were found to be consistent with those of studies for a number of proteins: GM-CSF (*CSF2*), LIF (*LIF*), Osteopontin (*SPP1*), GROa (*CXCL1*), G-SCF (*CSF3*), and FGF2 (*FGF2*). Some genes and proteins involved in a number of processes, including the proliferation of myoblasts (*IGF1*, FGF7, HGF), hepatocytes (*MDK*, *PTN*), endotheliocytes (*PGF*, *MDK*, ANG, FGF7), epithelial cells (*PGF*, *IGF1*, *MDK*, *PTN*, ANG, FGF7), and other cell populations, were decreased. The data obtained indicate a shift in the balance of secretion toward a more inflammatory one, which is characteristic of SASP. On the other hand, they allow for the characterization of the SASP of MmC-treated ASC52telo in more detail.

### 3.2. EC Analysis (Effects of Cocultivation and CM of Senescent MSCs)

The subsequent stage of the study was dedicated to the analysis of the response of ECs to direct cocultivation with senescent MSCs (MmC+), as well as under the influence of their secretome (CM). As a control, we employed a monoculture of ECs cultivated in a standard growth medium or a coculture of ECs with intact MSCs (MmC−). To characterize ECs, cell separation was performed after cocultivation. The cell count, non-directional migration, activity of cellular organelles, ROS level, activation marker ICAM-1, expression of aging genes *P16* and *P21*, pro-inflammatory cytokine genes *IL6* and *IL8*, and integrin subunit genes VLA-1 were assessed in order to ascertain the impact of the experimental conditions on the cells. The conditioned medium was also analyzed after 48 h of the experiment.

**Figure 6:**
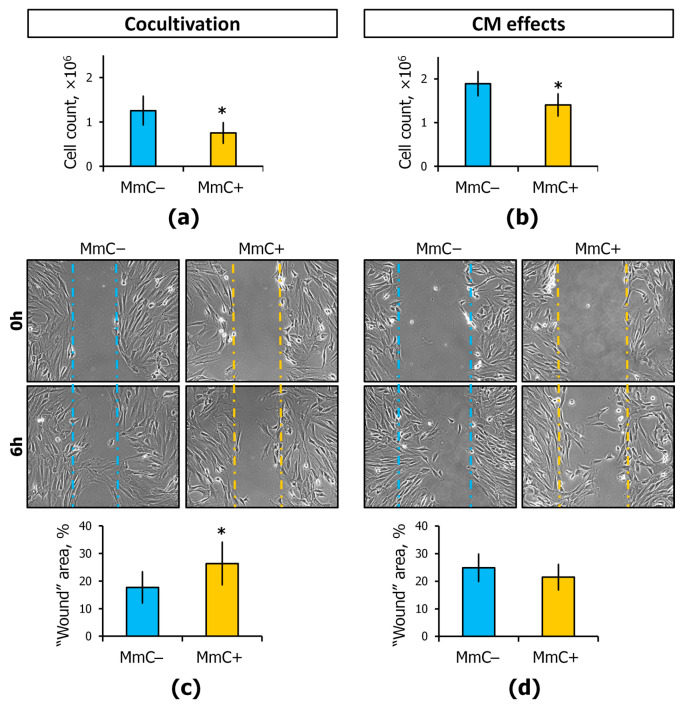
Cell count and “wound healing”. The effects of direct cocultivation with senescent MSCs or conditioned medium (CM) from senescent MSCs: (**a**,**b**) the cell growth, count of ECs; (**c**,**d**) “wound healing” assay, EC migration. Representative images of “wound healing” (light microscopy) after 6 h scraping and “wound” area (%, initial “wound” area = 100%) are shown. Data are shown as mean ± SD; *n* ≥ 4, * *p* < 0.05. MmC—mitomycin C, MSCs—mesenchymal stem cells, ECs—endothelial cells.

The proliferation and migration of ECs are essential for vascular remodeling. ECs migrate during vasculogenesis and angiogenesis, but they also migrate in a damaged vessel to restore vessel integrity. The process is modulated by a complex network of signaling, intercellular signals, and environmental cues [32,33]. In coculture with MSCs (MmC+), a significant decrease in EC count by 40% was observed (Figure 6a). The addition of CM from senescent MSCs to ECs resulted in a similar effect, with cell count being reduced by 30% (Figure 6b). It is likely that the growth of ECs is largely influenced by paracrine mediators and not by direct contact interaction. The viability of the ECs remained above 95% throughout the experimental period. It can be reasonably concluded that the observed changes in EC count were due to changes in proliferation.

The non-targeted migration of ECs or coculture ECs-MSCs was evaluated in cell monolayers. A decrease in migratory activity was observed in coculture with MmC-treated MSCs, as the wound area decreased more slowly (1.6 times) (Figure 6c and Figure S1a). However, in a monoculture of ECs with the addition of CM, no similar effect was observed (Figure 6d and Figure S1b).

**Figure 7:**
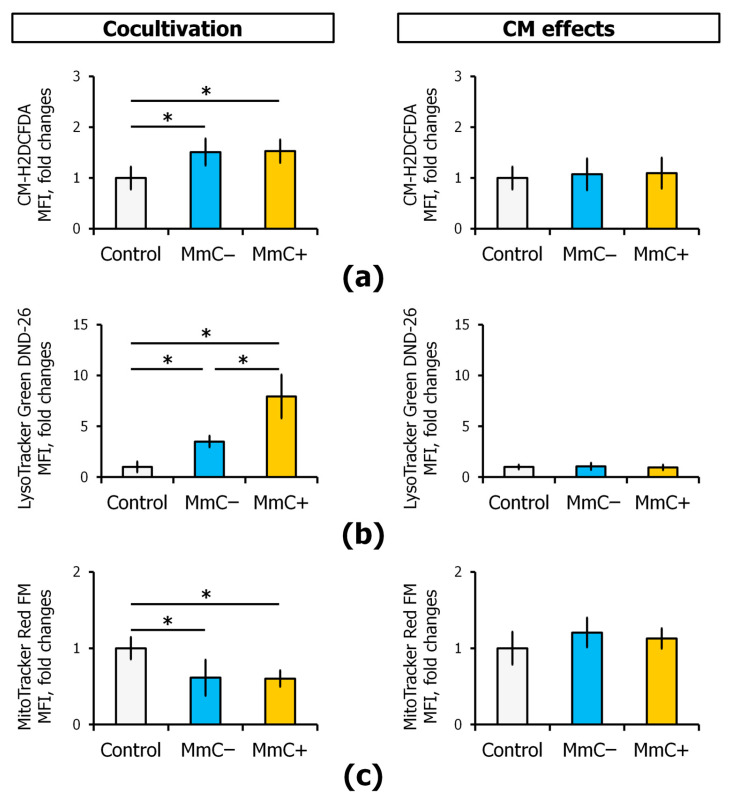
Analysis of the intracellular compartment activity and the oxidative stress level of ECs. The effects of direct cocultivation with senescent MSCs or conditioned medium (CM) from senescent MSCs: (**a**) the intracellular ROS, determined by CM-H2DCFDA staining; (**b**) the lysosomal compartment activity assessed using the analysis of LysoTracker Green DND-26; (**c**) the mitochondrial compartment activity assessed using the analysis of MitoTracker Red FM. Flow cytometry. Data are shown as mean ± SD; *n* ≥ 4, * *p* < 0.05. MmC—mitomycin C, MSCs—mesenchymal stem cells, ECs—endothelial cells.

Subsequently, the level of ROS and the activity of the lysosomal and mitochondrial compartments in ECs were evaluated. It has been demonstrated that interaction with MSCs (both MmC− and MmC+) results in an increase in ROS levels (Figure 7a). However, the specific effects of senescent cells have not been revealed. A multitude of studies have documented the role of oxidative stress in the aging process. In particular, several models suggest that ROS modulates age-related pathologies. The damage of biological macromolecules caused by oxidative stress is a significant contributing factor to the development of numerous age-related chronic diseases [34].

To investigate the lysosomal compartment activity, the accumulation of LysoTracker Green DND-26 in intracellular vesicles with low pH was examined. The activity of the lysosomal compartment in ECs was found to increase by 3.5 times when interacting with “young” MSCs and by eight times when interacting with senescent cells (Figure 7b). Previously, we demonstrated an increased LysoTracker fluorescence in replicative senescent MSCs [35,36]. Some experiments demonstrated an elevated LysoTracker fluorescence after different stress conditions [37]. Interaction with MSCs (both MmC− and MmC+) leads to a decrease in the activity of the mitochondrial compartment in ECs (Figure 7c).

The analysis of the activity of intracellular compartments and the level of oxidative stress in ECs under the influence of the secretome of MSCs did not reveal any significant changes in the functional activity of lysosomes, mitochondria, and ROS levels in any of the comparison groups. (Figure 7a–c).

**Figure 8:**
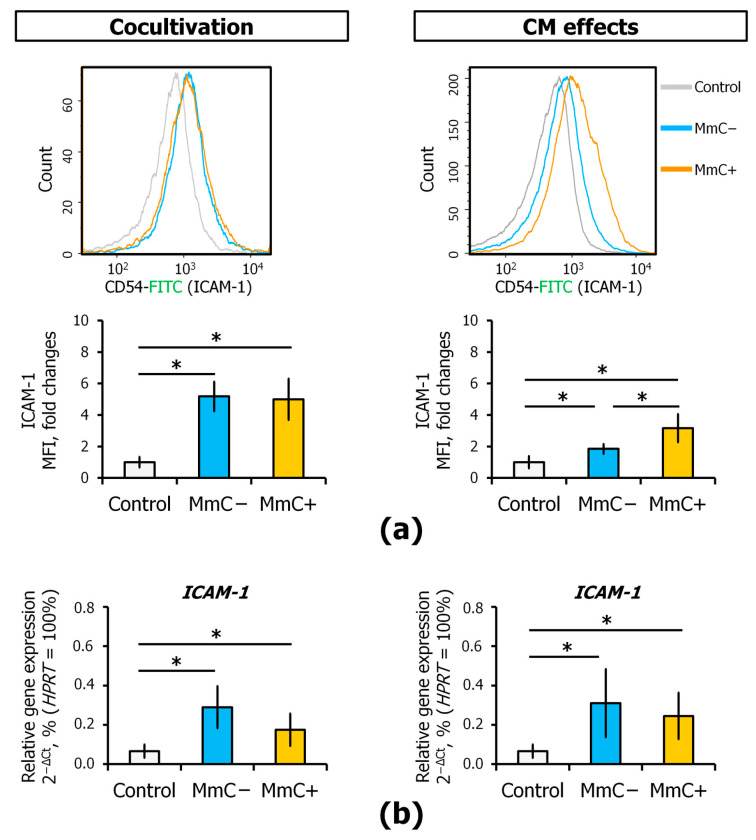
The expression of ICAM-1 in ECs. The effects of direct cocultivation with senescent MSCs or conditioned medium (CM) from senescent MSCs: (**a**) expression of ICAM1 receptor evaluated using flow cytometry analysis; (**b**) differential expression of *ICAM1* gene evaluated using q-PCR analysis. Data are shown as mean ± SD; *n* ≥ 4, * *p* < 0.05. MmC—mitomycin C, MSCs—mesenchymal stem cells, ECs—endothelial cells, Control—untreated ECs.

The stimulation by specific agents results in endothelial cell activation, which allows the endothelium to participate in the inflammatory response. This process is characterized by a series of stereotyped events, although the resulting effects are diverse. Nevertheless, all of these effects interact with each other to cause local inflammation [38]. The expression of an EC activation marker on the cell membrane was assessed using flow cytometry. The interaction of ECs with MSCs (both MmC±) resulted in a significant fivefold increase in intercellular adhesion molecule 1 (ICAM-1) expression (Figure 8a). The addition of CM also increased the expression of ICAM-1, but the effect was less pronounced than in coculture. Notably, the secretome derived from senescent MSCs exhibited a more pronounced effect than that of “young” MSCs (Figure 8a). At the transcriptional level, an increase in *ICAM-1* gene expression was also observed in response to direct interaction with MSCs or to the addition of CM (Figure 8b).

**Figure 9:**
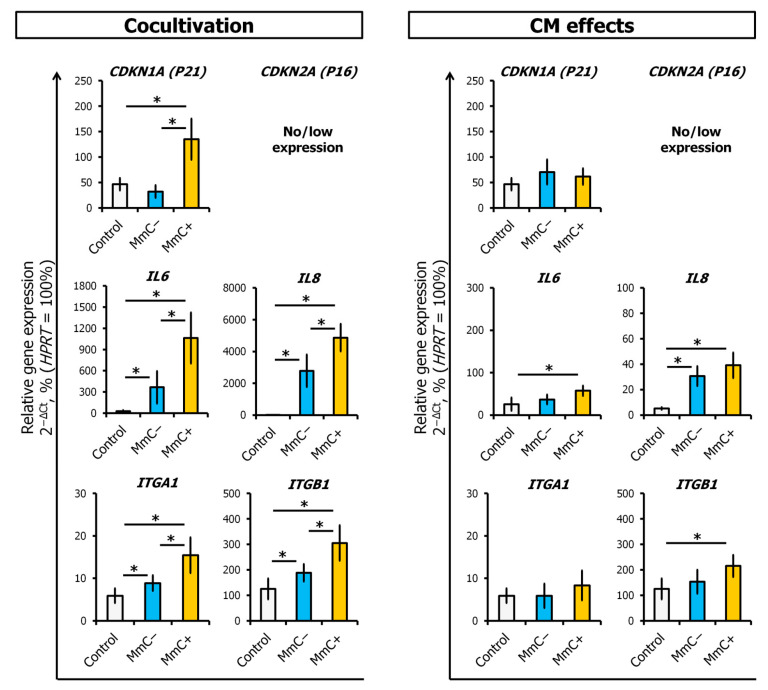
The differential gene expression of *CDKN1A* (*P21*), *IL-6*, *IL-8*, *ITGA1*, and *ITGB1* in ECs evaluated using q-PCR analysis. The effects of direct cocultivation with senescent MSCs or conditioned medium (CM) from senescent MSCs. Data are shown as mean ± SD; *n* ≥ 4, * *p* < 0.05. MmC—mitomycin C, MSCs—mesenchymal stem cells, ECs—endothelial cells, Control—untreated ECs.

We conducted further examinations of the expression of several genes in ECs, including *CDKN1A* (*P21*), *IL6*, *IL8*, and *ITGA1*, *ITGB1*. The expression of *CDKN1A* (*P21*) is regulated by the tumor suppressor protein P53, which mediates the P53-dependent cell cycle G1 phase arrest in response to a variety of stress inducers. This protein can interact with PCNA, a DNA polymerase accessory factor, and plays a regulatory role in S phase DNA replication and DNA damage repair. IL-6 and IL-8 are important pro-inflammatory components of the SASP. The α1β1 integrin (*ITGA1* and *ITGB1* encoded), also known as VLA-1, interacts with laminin and collagen in the extracellular matrix (ECM). By means of transmembrane signaling, it mediates adhesion, migration, proliferation, remodeling of the extracellular matrix (ECM), and cytokine secretion by endothelial cells and immunocytes. Of note, its expressions and functions are enhanced by inflammatory cytokines [39]. Upon interaction with senescent MSCs, there was a notable increase in the expression of all the studied genes. Interestingly, *CDKN1A* expression did not respond to interaction with “young” MSCs (Figure 9, left). When CM was added without direct contact with cells, the effects were weaker. However, SASP resulted in the upregulation of *IL6*, *IL8*, and *ITGB1* (Figure 9, right).

In light of the comprehensive data set, it can be reasonably concluded that the direct interaction of ECs with senescent MSCs results in more pronounced alterations in their functional state when compared to the effects of the MSC secretome. It is important to note that the short exposures considered in this study do not allow for the detection of long-term changes. Nevertheless, alterations have been observed at the transcriptional level, which could potentially result in a changed state of ECs, including activation and maintenance of the inflammatory response induced by senescent MSCs and the products of their paracrine activity in the microenvironment.

### 3.3. CM Analysis

**Figure 10:**
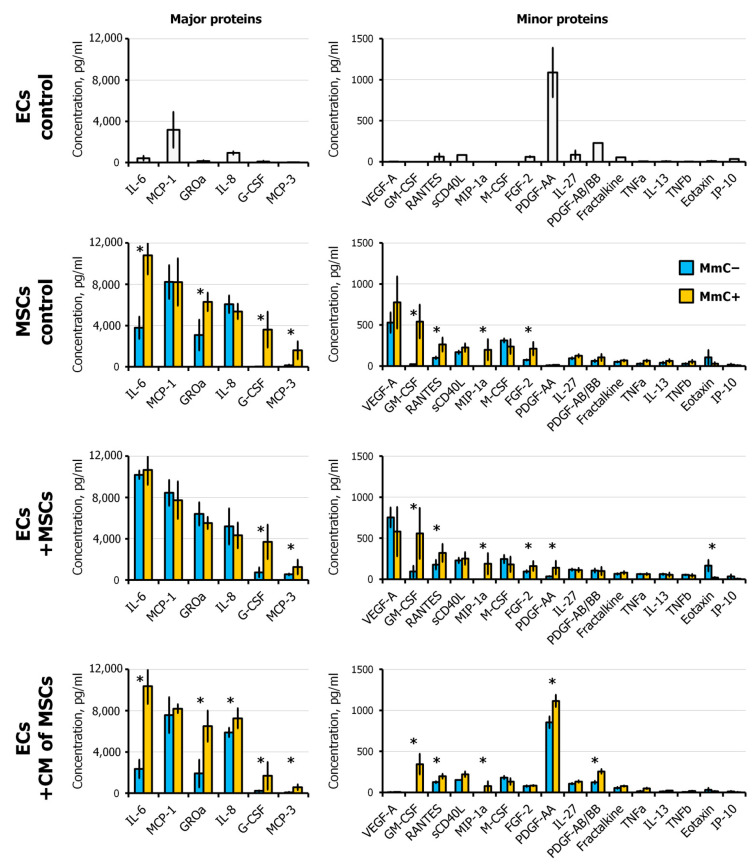
Cytokine concentration in conditioned medium of ECs and MSCs in monoculture, ECs cocultured with MSCs and ECs under conditioned medium from MSCs, evaluated using multiplexed fluorescent bead-based immunoassay. Data are shown as mean ± SD; *n* ≥ 4, * *p* < 0.05. MmC—mitomycin C, MSCs—mesenchymal stem cells, ECs—endothelial cells, EC control—monoculture of ECs (untreated), MSC control—monoculture of MSCs (MmC− and MmC+), ECs + MSCs—coculture, ECs + CM of MSCs—CM-treated ECs.

Ten days after exposure to MmC, MSCs were subcultured into monoculture (MSC control) and into coculture with ECs (ECs + MSCs). In parallel, a monoculture of ECs (EC control) and a culture of ECs with the addition of CM from MSCs (ECs + CM of MSCs) were established. Analysis of the conditioned medium in all samples was carried out after 48 h (Figure 10).

Upon analysis of the paracrine profile of the CM from the EC/MSC(MmC±) coculture, it was observed that the predominant MSC activity was present. Interestingly, the concentrations of IL-6 and GROa did not differ between the cocultures with MmC− and MmC+ MSCs. Conversely, in the MSC monoculture, the level of these cytokines was significantly higher in MmC+ cells. It is probable that the presence of endothelial cells prompts MSCs to secrete these cytokines, yet the upper limit of concentration is constrained by feedback loops. Consequently, the simultaneous influence of two factors (interaction with the endothelium and senescence) does not result in a summation of effects.

In comparison with the EC/MSC(MmC-) group, the level of G-CSF (5 times), GM-CSF (6 times), MCP-3 (2.3 times), RANTES, MIP-1α, and FGF-2 in the EC/MSC(MmC+) coculture was significantly increased. Conversely, the concentration of Eotaxin was significantly decreased (8.3 times). The majority of secreted mediators correspond to the profile of senescent MSCs. Of particular interest is PDGF-AA. This cytokine is almost not detected in the MSC monoculture but is present in large quantities in the EC monoculture. Interestingly, cell–cell interaction significantly reduces the content of PDGF-AA in the CM. At the same time, in CM with senescent MSCs, its level was higher. PDGFs are growth factors that promote cell proliferation and migration. Moreover, PDGFs have been demonstrated to regulate cell differentiation [40]. These proteins bind and activate PDGF receptor tyrosine kinases, which play a role in a wide range of developmental processes.

It was shown that in response to a cutaneous wound, senescent fibroblasts and endothelial cells secrete PDGF-AA, which facilitates wound closure by inducing myofibroblast differentiation. In two mouse models, the topical treatment of senescence-free wounds with recombinant PDGF-AA rescued the delayed wound closure and lack of myofibroblast differentiation. The results indicate that the SASP may play a beneficial role in tissue repair [41].

In EC monoculture, the addition of “senescent” CM results in an increased content of G-CSF, GM-CSF, GROa, IL-6, IL-8, MCP-3, RANTES, MIP-1, and PDGF-AA. It is noteworthy that PDGF-AA is not detected in MSC monoculture and is secreted only by EC. This implies that the addition of the senescent secretome from MSCs increases the production of PDGF-AA by ECs. The data also indicate that the senescent secretome of MSCs enhances the production of the inflammatory factor IL-8 in ECs. The concentration of VEGF was completely decreased in the conditioned medium. It was shown earlier in other works. It was hypothesized that VEGF may be metabolized by endothelial cells to accelerate the formation of vascular structures [42].

## 4. Discussion

Cell senescence plays a beneficial role in regulating a number of biological processes, including embryonic development, wound healing, resolution of fibrosis, and tumor suppression. Nevertheless, prolonged senescence can result in a number of adverse effects, including the development of tumors, chronic inflammation, immune deficiency, and a reduction in the number of stem cells. Consequently, these alterations give rise to a number of age-related diseases, including osteoporosis, atherosclerosis, type 2 diabetes mellitus, metabolic syndrome, neurodegeneration, and cancer, among others [31]. In our work, we studied the effect of senescent MSCs on some functional characteristics of ECs.

The study of cellular and subcellular processes necessitates the use of appropriate in vitro models. Primary MSCs are subject to a number of limitations, including high variability between donors and tissues [43]. These issues have the potential to compromise the replicability of results across different scientific groups. One potential solution is the utilization of immortalized lines derived from primary human MSCs. In our study, we employed the ASC52telo line, derived from MSCs isolated from human adipose tissue. The line is immortalized through the introduction of the telomerase gene (hTERT). hTERT-immortalized MSCs have recently gained prominence in biological and medical research as replacements for primary MSCs. These cells have been employed in investigations into the role of MSCs in maintaining tissue homeostasis [44,45,46] and in the fabrication of scaffolds for tissue engineering [47]. To assess the regulatory potential of senescent MSCs, the endothelial line EA.hy926 was utilized. EA.hy926 cells have been extensively employed in a multitude of investigations pertaining to ECs [48,49,50]. These cells preserve similar characteristics to primary human ECs [51,52].

Similar to other cells, ASC52telo displays typical signs of cellular senescence under sublethal stress, including proteome and secretome shifts. As with other authors, we have observed this phenomenon in both present and previous studies [53,54]. Genotoxic agents, such as elevated ROS, ionizing radiation, or MmC, induce the upregulation of genes associated with senescence in immortalized fibroblasts and cancer cells. The activation of telomerase does not prevent stress-induced senescence [55,56,57].

The process of senescence is initiated in response to a variety of signals. A significant body of research has demonstrated that treatment with certain chemotherapeutic drugs and ionizing radiations provoke cell cycle arrest in cancer-and immortalized cells. Recent studies have demonstrated that treatment with some chemotherapeutic drugs and ionizing radiations provoke “therapy-induced senescence (TIS)” in cancer cells. Gorgoulis et al. include MmC in the “Selected List of Factors Triggering Senescence” as a DNA cross-linker. Genotoxic stimuli cause random damage to cellular macromolecules, leading to variation in the aging phenotype despite a common program. Following the activation of P16 or P21, cells progress to full senescence by downregulating lamin B1, thereby triggering extensive chromatin remodeling that underlies the production of a SASP, including pro-inflammatory cytokines, various growth factors, and proteases that collectively alter tissue structure and function. The SASP represents a defining characteristic that distinguishes senescent cells from other non-proliferating cells, including quiescent cells and terminally differentiated cells [2,30,58].

The arrest of the cell cycle in senescence is largely mediated via the activation of either one or both of the P53/P21 and P16/pRB tumor suppressor pathways. These pathways maintain the senescence state mainly by inducing alterations in gene expression, as P53 and pRB are key transcriptional regulators. The prolonged overexpression of any of the four critical components (P53, pRB, P16, P21) is sufficient to induce senescence [30,59,60]. In the context of our experimental conditions, the process of cellular senescence was observed to occur through the activation of P21. It is probable that the P16 pathway was not activated in both lines that were studied.

Following the process of senescence activation, cells exhibit SASP, which promotes the alteration of neighboring tissue microenvironments. Such SASP factors have been demonstrated to drive the mechanisms underlying the pleiotropic features of cellular senescence. The physiological and pathological roles of senescence beyond tumor suppression have been increasingly recognized. These include involvement in embryogenesis, tissue and organ aging, and wound healing. SASP can facilitate the regeneration of tissues through the stimulation of nearby progenitor cells. However, in certain situations, cell senescence has been demonstrated to have a tumor-promoting role [2,30,60].

In the course of our experiments, we observed the response of ECs to senescent MSCs. Our findings indicated a decrease in cellular expansion of ECs and migratory activity of coculture (ECs with senescent MSCs). It was previously established that the disruption of the formation of intercellular connections, suppression of cell growth and proliferation, and a decrease in migratory activity can cause dysfunction of the endothelial barrier [61]. In senescent cells, the impairment of cell–cell adhesion results in a disruption of endothelial barrier function [62]. The results demonstrated that soluble factors secreted by senescent MSCs and direct contact with senescent MSCs can have a negative effect on “young” ECs in the context of their regenerative potential. This is evidenced by a decrease in proliferative and migratory activities. Interestingly, 3-h MmC treatment reduces migration and deposition of TGFβ1-stimulated collagen by primary and hTERT-immortalized human corneal limbal epithelial [57].

Upon direct contact with senescent MSCs, an increase in the activity of the lysosomal compartment was observed. However, the SASP did not exert a similar influence. Such changes are characteristic of senescent cells or cells exposed to stress [17,36,37]. It is probable that alterations in lysosomal activity may be associated with augmented autophagic processes in response to stress-induced damage. On the other hand, when investigating the degree of oxidative stress, we did not observe an increase in ROS, which are frequently the primary destructive agents within the cell. We assume that 48 h after the initiation of the interaction, the level of ROS may return to normalcy due to the activation of antioxidant systems. However, the level of ROS remains elevated relative to the control group without the addition of MSCs. Meanwhile, the lysosomal compartment displays heightened activity and persists in utilizing damaged cellular elements.

Furthermore, it was observed that SASP causes an increase in the expression of ICAM-1 on the EC membrane. ICAM-1 is a master regulator of cell reaction in inflammation, tissue damage, and tumorigenesis [63]. Moreover, direct interaction with senescent cells led to a similar robust increase as with young ones. This is likely directly related to the content of pro-inflammatory cytokines in the microenvironment. In a direct interaction, IL-6 levels were found to be equally high in both cultures. When exposed to the secretome at 48 h, IL-6 and IL-8 were increased in a conditioned medium supplemented with SASP but not in the “young” secretome (Figure 10). ICAM-1 is a major adhesion protein that enables leukocytes and lymphocytes to adhere to vascular ECs. ICAM-1 is normally expressed at low levels on ECs, lymphocytes, and monocytes. However, its expression can be significantly increased in the presence of inflammatory cytokines. In addition to its intercellular adhesion properties, the interaction of ICAM-1 with cytoskeletal elements triggers important intracellular signaling events. This protein increases the activity of the transcription factor NF-κB, which in turn is a positive regulator of the inflammatory response [63,64]. Consequently, we observe the formation of a positive feedback loop. The elevated expression of VLA-1 integrin subunits (ITGA1 and ITGB1) also suggests that EC is undergoing pro-inflammatory activation.

It has been demonstrated that alterations in the regulation of ICAM-1 occur in both acute and chronic diseases. Previous studies have indicated that the expression of ICAM-1 and VCAM-1 on the surface of endothelial cells is closely associated with the formation of vascular diseases such as early atherosclerotic lesions. Furthermore, elevated ICAM-1 expression has been linked to ischemia in older adults [65,66]. The results of microarray analysis indicate that ICAM-1 overexpression is dependent on P53 during cellular senescence [58]. Given the pivotal role of ICAM-1 expression and signaling in regulating plasma membrane function, it is plausible that this molecule serves as a pivotal transporter, contributing to the observed alterations in membrane function associated with the aging process [67]. It has been recently demonstrated in vitro that EC senescence results in significant alterations in the expression and functionality of ICAM-1, which in turn gives rise to changes in signal transduction [68]. It is probable that the increase in ICAM-1 expression observed in our model occurred independently of the P53/P21 pathway, potentially due to the brief exposure period (48 h).

Endothelial dysfunction is primarily the result of the persistence of a pro-inflammatory microenvironment. Indeed, the aging process is associated with a progressive tendency toward low-grade chronic inflammation, which is referred to as “inflammaging”. This phenomenon correlates with the rate of aging and the risk of developing major degenerative diseases in older adults [69,70]. This physiological state is characterized by increased levels of pro-inflammatory cytokines, including IL-6 and TNF-α. Senescent MSCs release SASP factors, which paracrinely transmit inflammatory signals to neighboring ECs, potentially contributing to a decrease in proliferative activity and the acquisition of a pro-inflammatory phenotype. The observed outcomes may suggest that a pro-inflammatory cocktail secreted by senescent MSCs may contribute to the disruption of endothelial barrier integrity.

The results of the PCR analysis indicated that the interaction of senescent MSCs with ECs led to the increased expression of the pro-inflammatory cytokines *IL6* and *IL8*, as well as integrin subunits responsible for increased adhesion to the extracellular matrix (ECM) directly in the ECs themselves. The direct interaction led to much more pronounced effects. The results also demonstrated increased expression of the *P21* gene, which may indicate intracellular damage and is involved in senescence processes. These data provide support for the hypothesis that cellular senescence can spread to neighboring cells. Notably, these data were obtained on immortalized cells that are incapable of replicative senescence.

Analyses of primary MSCs obtained from human bone marrow yield comparable results. The secretion of factors by senescent MSCs results in the activation of pro-inflammatory gene expression in young hematopoietic stem cells, accompanied by a reduction in their clonogenic potential [71]. In our previous study, we seeded ECs on a decellularized ECM obtained from senescent MSCs. We observed a change in the morphology of the cell layer and a decrease in cell proliferation. Additionally, we noted an increase in the production of the inflammatory chemokines MCP1 and GROα and a decrease in the proangiogenic growth factor FGF-2 [72]. It is plausible that this paracrine mechanism may also operate in the reverse direction. A recent study demonstrated that microvesicles derived from “young” MSCs can enhance the condition of senescent MSCs, reducing the intensity of senescence-associated signs. This includes a decline in the activity of the SA-β-gal enzyme, the expression of *P21* and *P53*, as well as the production of pro-inflammatory cytokines. The proteome of microvesicles was analyzed, and it was found that they are rich in antioxidant enzymes, specifically peroxiredoxins. This may partly explain their “rejuvenating” effect [73]. The administration of extracellular vesicles derived from adipose-derived MSCs to mice resulted in the induction of pro-regenerative effects and a reduction in oxidative stress, inflammation, and senescence markers in both muscle and kidney tissue [74].

The SASP comprises pro-inflammatory cytokines, growth factors, and extracellular matrix-degrading proteins. It is hypothesized that this complex network of molecules evolved as a means for developing cells to communicate with the immune system but also as an extracellular signal to promote the regeneration of tissues through the stimulation of nearby progenitor cells [2]. Nevertheless, it has been demonstrated that a “chronic” SASP is capable of inducing senescence in adjacent young cells, thereby contributing to tissue dysfunction [75] and, paradoxically, tumorigenesis [41]. It has been postulated that an “acute” type of senescence plays a generally beneficial role in processes such as embryonic development and wound healing. Conversely, a “chronic” type of senescence may contribute to aging and age-related disease.

## 5. Conclusions

Our model, which comprised two types of cells, demonstrated that a 48 h direct interaction resulted in a reduction in the proliferative and migratory activity of ECs. Additionally, there was an increase in the activity of the lysosomal compartment and the expression of the *P21*, *IL6*, *IL8*, *ITGA1*, and *ITGB1* genes. The addition of SASP resulted in less pronounced effects. However, decreased proliferation and increased expression of ICAM-1 were observed. The addition of SASP from MSCs results in the maintenance of high levels of “senescent” cytokines and growth factors after 48 h. In contrast, direct interaction with MSCs suppresses the production of the “reparative” cytokine PDGF-AA by endothelial cells. Exposure to the secretome does not have this effect. Moreover, in samples with the addition of the “senescent” secretome, the level of PDGF-AA was higher, which may explain some of the pro-regenerative role of SASP in a number of experiments. The role of senescent cells in tissues remains controversial, but studying simple models with a limited number of cell–cell interactions allows us to learn more about the individual mechanisms underlying aging-associated diseases.

## Data Availability

Data are contained within the article.

## Supplementary Materials

The following supporting information can be downloaded at: https://www.mdpi.com/article/10.3390/cells13161345/s1, Figure S1: Images of “Wound healing”.

## References

[1] Muñoz-Espín D., Serrano M. (2014). Cellular senescence: From physiology to pathology. Nat. Rev. Mol. Cell Biol..

[2] Van Deursen J.M. (2014). The role of senescent cells in ageing. Nature.

[3] McHugh D., Gil J. (2018). Senescence and aging: Causes, consequences, and therapeutic avenues. J. Cell Biol..

[4] Lewis-McDougall F.C., Ruchaya P.J., Domenjo-Vila E., Shin Teoh T., Prata L., Cottle B.J., Clark J.E., Punjabi P.P., Awad W., Torella D. (2019). Aged-senescent cells contribute to impaired heart regeneration. Aging Cell.

[5] Sławińska N., Krupa R. (2021). Molecular aspects of senescence and organismal ageing—DNA damage response, telomeres, inflammation and chromatin. Int. J. Mol. Sci..

[6] Kuilman T., Peeper D.S. (2009). Senescence-messaging secretome: SMS-ing cellular stress. Nat. Rev. Cancer.

[7] Coppé J.P., Desprez P.Y., Krtolica A., Campisi J. (2010). The senescence-associated secretory phenotype: The dark side of tumor suppression. Annu. Rev. Pathol. Mech. Dis..

[8] Nelson G., Kucheryavenko O., Wordsworth J., von Zglinicki T. (2018). The senescent bystander effect is caused by ROS-activated NF-κB signalling. Mech. Ageing Dev..

[9] Kumar M., Yan P., Kuchel G.A., Xu M. (2024). Cellular Senescence as a Targetable Risk Factor for Cardiovascular Diseases: Therapeutic Implications: JACC Family Series. Basic Transl. Sci..

[10] Lunyak V.V., Amaro-Ortiz A., Gaur M. (2017). Mesenchymal stem cells secretory responses: Senescence messaging secretome and immunomodulation perspective. Front. Genet..

[11] Pittenger M.F., Discher D.E., Péault B.M., Phinney D.G., Hare J.M., Caplan A.I. (2019). Mesenchymal stem cell perspective: Cell biology to clinical progress. NPJ Regen. Med..

[12] Kehl D., Generali M., Mallone A., Heller M., Uldry A.C., Cheng P., Gantenbein B., Hoerstrup S.P., Weber B. (2019). Proteomic analysis of human mesenchymal stromal cell secretomes: A systematic comparison of the angiogenic potential. NPJ Regen. Med..

[13] Holnthoner W., Hohenegger K., Husa A.M., Muehleder S., Meinl A., Peterbauer-Scherb A., Redl H. (2015). Adipose-derived stem cells induce vascular tube formation of outgrowth endothelial cells in a fibrin matrix. J. Tissue Eng. Regen. Med..

[14] Mohamad Yusoff F., Higashi Y. (2023). Mesenchymal Stem/Stromal Cells for Therapeutic Angiogenesis. Cells.

[15] Turinetto V., Vitale E., Giachino C. (2016). Senescence in human mesenchymal stem cells: Functional changes and implications in stem cell-based therapy. Int. J. Mol. Sci..

[16] Legzdina D., Romanauska A., Nikulshin S., Kozlovska T., Berzins U. (2016). Characterization of senescence of culture-expanded human adipose-derived mesenchymal stem cells. Int. J. Stem Cells.

[17] Ratushnyy A.Y., Buravkova L.B. (2020). Cell senescence and mesenchymal stromal cells. Hum. Physiol..

[18] Campisi J., d’Adda di Fagagna F. (2007). Cellular senescence: When bad things happen to good cells. Nat. Rev. Mol. Cell Biol..

[19] Blagosklonny M.V. (2006). Aging and immortality: Quasi-programmed senescence and its pharmacologic inhibition. Cell Cycle.

[20] Bertolo A., Baur M., Guerrero J., Pötzel T., Stoyanov J. (2019). Autofluorescence is a reliable in vitro marker of cellular senescence in human mesenchymal stromal cells. Sci. Rep..

[21] Dominici M.L.B.K., Le Blanc K., Mueller I., Slaper-Cortenbach I., Marini F.C., Krause D.S., Deans R.J., Keating A., Prockop D.J., Horwitz E.M. (2006). Minimal criteria for defining multipotent mesenchymal stromal cells. The International Society for Cellular Therapy position statement. Cytotherapy.

[22] Yurttas C., Hoffmann G., Tolios A., Haen S.P., Schwab M., Königsrainer I., Königsrainer A., Beckert S., Löffler M.W. (2018). Systematic review of variations in hyperthermic intraperitoneal chemotherapy (HIPEC) for peritoneal metastasis from colorectal cancer. J. Clin. Med..

[23] Chugh R.M., Chaturvedi M., Yerneni L.K. (2016). Exposure cell number during feeder cell growth-arrest by Mitomycin C is a critical pharmacological aspect in stem cell culture system. J. Pharmacol. Toxicol. Methods.

[24] Lee Y.J., Park S.J., Ciccone S.L., Kim C.R., Lee S.H. (2006). An in vivo analysis of MMC-induced DNA damage and its repair. Carcinogenesis.

[25] McKenna E., Traganos F., Zhao H., Darzynkiewicz Z. (2012). Persistent DNA damage caused by low levels of mitomycin C induces irreversible cell senescence. Cell Cycle.

[26] Dimri G.P., Lee X., Basile G., Acosta M., Scott G., Roskelley C., Medrano E.E., Linskens M., Rubelj I., Pereira-Smith O. (1995). A biomarker that identifies senescent human cells in culture and in aging skin in vivo. Proc. Natl. Acad. Sci. USA.

[27] Fingar D.C., Salama S., Tsou C., Harlow E.D., Blenis J. (2002). Mammalian cell size is controlled by mTOR and its downstream targets S6K1 and 4EBP1/eIF4E. Genes Dev..

[28] Lee S.S., Vũ T.T., Weiss A.S., Yeo G.C. (2023). Stress-induced senescence in mesenchymal stem cells: Triggers, hallmarks, and current rejuvenation approaches. Eur. J. Cell Biol..

[29] Ilie O.D., Ciobica A., Riga S., Dhunna N., McKenna J., Mavroudis I., Doroftei B., Ciobanu A.-M., Riga D. (2020). Mini-review on lipofuscin and aging: Focusing on the molecular interface, the biological recycling mechanism, oxidative stress, and the gut-brain axis functionality. Medicina.

[30] Kumari R., Jat P. (2021). Mechanisms of cellular senescence: Cell cycle arrest and senescence associated secretory phenotype. Front. Cell Dev. Biol..

[31] Huang W., Hickson L.J., Eirin A., Kirkland J.L., Lerman L.O. (2022). Cellular senescence: The good, the bad and the unknown. Nat. Rev. Nephrol..

[32] Michaelis U.R. (2014). Mechanisms of endothelial cell migration. Cell. Mol. Life Sci..

[33] Morbidelli L., Orlando C., Maggi C.A., Ledda F., Ziche M. (1995). Proliferation and migration of endothelial cells is promoted by endothelins via activation of ETB receptors. Am. J. Physiol.-Heart Circ. Physiol..

[34] Pole A., Dimri M., Dimri G.P. (2016). Oxidative stress, cellular senescence and ageing. AIMS Mol. Sci..

[35] Ratushnyy A.Y., Rudimova Y.V., Buravkova L.B. (2020). Replicative senescence and expression of autophagy genes in mesenchymal stromal cells. Biochemistry.

[36] Ratushnyy A., Lobanova M., Buravkova L.B. (2017). Expansion of adipose tissue-derived stromal cells at “physiologic” hypoxia attenuates replicative senescence. Cell Biochem. Funct..

[37] Yoon J., Bang S.H., Park J.S., Chang S.T., Kim Y.H., Min J. (2011). Increased in vitro lysosomal function in oxidative stress-induced cell lines. Appl. Biochem. Biotechnol..

[38] Ballermann B.J. (1998). Endothelial cell activation. Kidney Int..

[39] Ben-Horin S., Bank I. (2004). The role of very late antigen-1 in immune-mediated inflammation. Clin. Immunol..

[40] Li A., Xia X., Yeh J., Kua H., Liu H., Mishina Y., Hao A., Li B. (2014). PDGF-AA promotes osteogenic differentiation and migration of mesenchymal stem cell by down-regulating PDGFRα and derepressing BMP-Smad1/5/8 signaling. PLoS ONE.

[41] Demaria M., Ohtani N., Youssef S.A., Rodier F., Toussaint W., Mitchell J.R., Laberge R.-M., Vijg J., Van Steeg H., Dollé M.E. (2014). An essential role for senescent cells in optimal wound healing through secretion of PDGF-AA. Dev. Cell.

[42] Steiner D., Köhn K., Beier J.P., Stürzl M., Horch R.E., Arkudas A. (2017). Cocultivation of mesenchymal stem cells and endothelial progenitor cells reveals antiapoptotic and proangiogenic effects. Cells Tissues Organs.

[43] Elahi K.C., Klein G., Avci-Adali M., Sievert K.D., MacNeil S., Aicher W.K. (2016). Human mesenchymal stromal cells from different sources diverge in their expression of cell surface proteins and display distinct differentiation patterns. Stem Cells Int..

[44] Pitrone M., Pizzolanti G., Tomasello L., Coppola A., Morini L., Pantuso G., Ficarella R., Guarnotta V., Perrini S., Giorgino F. (2017). NANOG plays a hierarchical role in the transcription network regulating the pluripotency and plasticity of adipose tissue-derived stem cells. Int. J. Mol. Sci..

[45] Maj M., Kokocha A., Bajek A., Drewa T. (2018). The interplay between adipose-derived stem cells and bladder cancer cells. Sci. Rep..

[46] Kulebyakin K., Tyurin-Kuzmin P., Efimenko A., Voloshin N., Kartoshkin A., Karagyaur M., Grigorieva O., Novoseletskaya E., Sysoeva V., Makarevich P. (2021). Decreased insulin sensitivity in telomerase-immortalized mesenchymal stem cells affects efficacy and outcome of adipogenic differentiation in vitro. Front. Cell Dev. Biol..

[47] Zitnay J.L., Reese S.P., Tran G., Farhang N., Bowles R.D., Weiss J.A. (2018). Fabrication of dense anisotropic collagen scaffolds using biaxial compression. Acta Biomater..

[48] Kaissarian N., Kang J., Shu L., Ferraz M.J., Aerts J.M., Shayman J.A. (2018). Dissociation of globotriaosylceramide and impaired endothelial function in α-galactosidase-A deficient EA.hy926 cells. Mol. Genet. Metab..

[49] Kraehling J.R., Chidlow J.H., Rajagopal C., Sugiyama M.G., Fowler J.W., Lee M.Y., Zhang X., Ramírez C.M., Park E.J., Tao B. (2016). Genome-wide RNAi screen reveals ALK1 mediates LDL uptake and transcytosis in endothelial cells. Nat. Commun..

[50] Yu F., Fu R., Liu L., Wang X., Wu T., Shen W., Gui Z., Mo X., Fang B., Xia L. (2019). Leptin-induced angiogenesis of EA.Hy926 endothelial cells via the Akt and Wnt signaling pathways in vitro and in vivo. Front. Pharmacol..

[51] Ahn K., Pan S., Beningo K., Hupe D. (1995). A permanent human cell line (EA.hy926) preserves the characteristics of endothelin converting enzyme from primary human umbilical vein endothelial cells. Life Sci..

[52] Thornhill M.H., Li J., Haskard D.O. (1993). Leucocyte endothelial cell adhesion: A study comparing human umbilical vein endothelial cells and the endothelial cell line EA-hy-926. Scand. J. Immunol..

[53] Comas F., Latorre J., Ortega F., Oliveras-Cañellas N., Lluch A., Ricart W., Fernández-Real J.M., Moreno-Navarrete J.M. (2021). Permanent cystathionine-β-Synthase gene knockdown promotes inflammation and oxidative stress in immortalized human adipose-derived mesenchymal stem cells, enhancing their adipogenic capacity. Redox Biol..

[54] Matveeva D., Kashirina D., Ezdakova M., Larina I., Buravkova L., Ratushnyy A. (2024). Senescence-Associated Alterations in Matrisome of Mesenchymal Stem Cells. Int. J. Mol. Sci..

[55] De Magalhães J.P., Chainiaux F., Remacle J., Toussaint O. (2002). Stress-induced premature senescence in BJ and hTERT-BJ1 human foreskin fibroblasts. FEBS Lett..

[56] Gorbunova V., Seluanov A., Pereira-Smith O.M. (2003). Evidence that high telomerase activity may induce a senescent-like growth arrest in human fibroblasts. J. Biol. Chem..

[57] Pal-Ghosh S., Tadvalkar G., Lieberman V.R., Guo X., Zieske J.D., Hutcheon A., Stepp M.A. (2019). Transient Mitomycin C-treatment of human corneal epithelial cells and fibroblasts alters cell migration, cytokine secretion, and matrix accumulation. Sci. Rep..

[58] Gorgoulis V., Adams P.D., Alimonti A., Bennett D.C., Bischof O., Bishop C., Campisi J., Collado M., Evangelou K., Ferbeyre G. (2019). Cellular senescence: Defining a path forward. Cell.

[59] McConnell B.B., Starborg M., Brookes S., Peters G. (1998). Inhibitors of cyclin-dependent kinases induce features of replicative senescence in early passage human diploid fibroblasts. Curr. Biol..

[60] Kobashigawa S., Sakaguchi Y.M., Masunaga S., Mori E. (2019). Stress-induced cellular senescence contributes to chronic inflammation and cancer progression. Therm. Med..

[61] Zheng H., Huang Q., Huang S., Yang X., Zhu T., Wang W., Wang H., He S., Ji L., Wang Y. (2018). Senescence inducer shikonin ROS-dependently suppressed lung cancer progression. Front. Pharmacol..

[62] Krouwer V.J., Hekking L.H., Langelaar-Makkinje M., Regan-Klapisz E., Post J.A. (2012). Endothelial cell senescence is associated with disrupted cell-cell junctions and increased monolayer permeability. Vasc. Cell.

[63] Bui T.M., Wiesolek H.L., Sumagin R. (2020). ICAM-1: A master regulator of cellular responses in inflammation, injury resolution, and tumorigenesis. J. Leukoc. Biol..

[64] Rahman A., Anwar K.N., Malik A.B. (2000). Protein kinase C-ζ mediates TNF-α-induced ICAM-1 gene transcription in endothelial cells. Am. J. Physiol.-Cell Physiol..

[65] Morisaki N., Saito I., Tamura K., Tashiro J., Masuda M., Kanzaki T., Watanabe S., Masuda Y., Saito Y. (1997). New indices of ischemic heart disease and aging: Studies on the serum levels of soluble intercellular adhesion molecule-1 (ICAM-1) and soluble vascular cell adhesion molecule-1 (VCAM-1) in patients with hypercholesterolemia and ischemic heart disease. Atherosclerosis.

[66] Thomas A.J., Ferrier I.N., Kalaria R.N., Davis S., O’Brien J.T. (2002). Cell adhesion molecule expression in the dorsolateral prefrontal cortex and anterior cingulate cortex in major depression in the elderly. Br. J. Psychiatry.

[67] Swift M.E., Burns A.L., Gray K.L., DiPietro L.A. (2001). Age-related alterations in the inflammatory response to dermal injury. J. Investig. Dermatol..

[68] Hubbard A.K., Rothlein R. (2000). Intercellular adhesion molecule-1 (ICAM-1) expression and cell signaling cascades. Free Radic. Biol. Med..

[69] Franceschi C., Bonafè M., Valensin S., Olivieri F., De Luca M., Ottaviani E., De Benedictis G. (2000). Inflamm-aging: An evolutionary perspective on immunosenescence. Ann. N. Y. Acad. Sci..

[70] Rea I.M., Gibson D.S., McGilligan V., McNerlan S.E., Alexander H.D., Ross O.A. (2018). Age and age-related diseases: Role of inflammation triggers and cytokines. Front. Immunol..

[71] Gnani D., Crippa S., Della Volpe L., Rossella V., Conti A., Lettera E., Rivis S., Ometti M., Fraschini G., Bernardo M.E. (2019). An early-senescence state in aged mesenchymal stromal cells contributes to hematopoietic stem and progenitor cell clonogenic impairment through the activation of a pro-inflammatory program. Aging Cell.

[72] Matveeva D.K., Ezdakova M.I., Ratushnyy A.Y. (2023). Modification of the Properties of Extracellular Matrix of Senescent Mesenchymal Stem Cells. Bull. Exp. Biol. Med..

[73] Liu S., Mahairaki V., Bai H., Ding Z., Li J., Witwer K.W., Cheng L. (2019). Highly purified human extracellular vesicles produced by stem cells alleviate aging cellular phenotypes of senescent human cells. Stem Cells.

[74] Sanz-Ros J., Romero-García N., Mas-Bargues C., Monleón D., Gordevicius J., Brooke R.T., Dromant M., Díaz A., Derevyanko A., Guío-Carrión A. (2022). Small extracellular vesicles from young adipose-derived stem cells prevent frailty, improve health span, and decrease epigenetic age in old mice. Sci. Adv..

[75] Acosta J.C., Banito A., Wuestefeld T., Georgilis A., Janich P., Morton J.P., Athineos D., Kang T.-W., Lasitschka F., Andrulis M. (2013). A complex secretory program orchestrated by the inflammasome controls paracrine senescence. Nat. Cell Biol..

