# Supplementary material for: Regulatory Effects of Senescent Mesenchymal Stem Cells: Endotheliocyte Reaction
Source: Cells. 2024 Aug 13;13(16):1345. doi: 10.3390/cells13161345 (PMC11352319; doi:10.3390/cells13161345)
Supplement: Supplementary file 1 [file cells-13-01345-s001.zip › cells-3123060-supplementary.pdf]

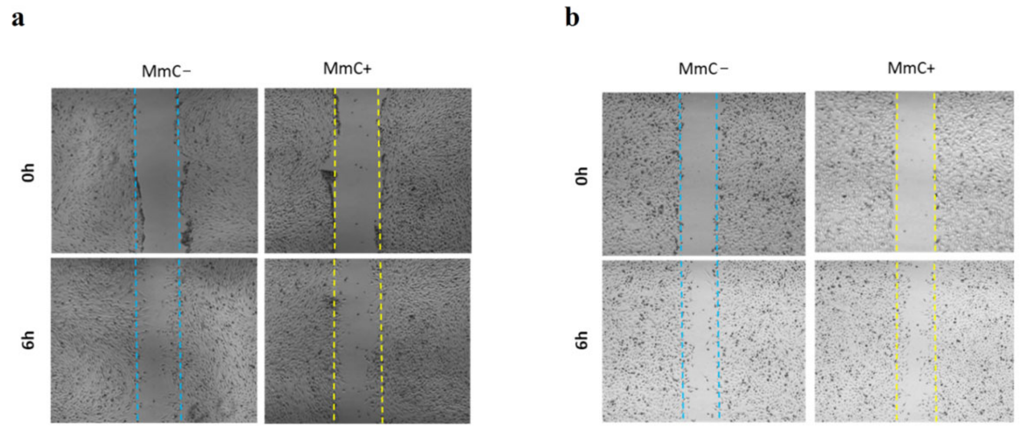

Figure S1. Images of "Wound healing" (microscopy, magnification x4): (**a**) The effects of direct cocultivation with senescent MSCs or (**b**) conditioned medium (CM) from senescent MSCs. MmC — mitomycin C.
